# Dual effects of targeting S100A11 on suppressing cellular metastatic properties and sensitizing drug response in gastric cancer

**DOI:** 10.1186/s12935-021-01949-1

**Published:** 2021-04-30

**Authors:** Yuxin Cui, Liting Li, Zhilei Li, Jie Yin, Jane Lane, Jiafu Ji, Wen G. Jiang

**Affiliations:** 1 Cardiff China Medical Research Collaborative, Cardiff University School of Medicine, Cardiff University, Heath Park, Cardiff, CF14 4XN UK; 2 China-Japan Friendship Hospital, Yinghuayuan East Street, Beijing, 10029 China; 3 Department of Pharmacy, Southern University of Science and Technology Hospital, Shenzhen, 518055 China; 4 Department of General Surgery, Beijing Friendship Hospital, Capital Medical University, Beijing, China; 5 Beijing Key Laboratory of Cancer Invasion and Metastasis Research and National Clinical Research Center for Digestive Diseases, 95 Yong-an Road, Xi-Cheng District, Beijing, 100050 China; 6 Key Laboratory of Carcinogenesis and Translational Research, Department of GI Surgery, Peking University Cancer Hospital and Institute, Beijing, 100142 China

**Keywords:** S100A11, Gastric cancer, Clinical significance, Drug resistance, Adhesion, Invasion

## Abstract

**Background:**

S100A11 is a member of the S100 family of proteins containing two EF-hand calcium-binding motifs. The dysregulated expression of the S100A11 gene has been implicated in tumour metastasis. However, the role of S100A11 protein in tumour cell response to chemotherapeutic drugs has not been characterised.

**Methods:**

Transcript levels of S100A11 in gastric cancer were evaluated using an in-house patient cohort. Protein expression of S100A11 in gastric cancer was estimated by immunohistochemistry of a tissue microarray. The stable gastric cancer cell lines were established using lentiviral shRNA vectors. The knockdown of S100A11 was validated by qRT-PCR, PCR, and Western blot. The cellular function of S100A11 was estimated by assays of cell adhesion, migration, and invasion. The cell cytotoxic assay was performed to investigate the response to chemotherapeutic drugs. An unsupervised hierarchical clustering and principal component analysis (HCPC) was applied to unveil the dimensional role of S100A11 among all S100 family members in gastric cancer.

**Results:**

High expression of S100A11 is associated with poor survival of gastric cancer patients (p < 0.001, HR = 1.85) and is an independent prognostic factor of gastric cancer. We demonstrate that S100A11 plays its role as a tumour promoter through regulating the MMP activity and the epithelial-mesenchymal transition (EMT) process. The stable knockdown of S100A11 suppresses the metastatic properties of gastric cancer cells, which include enhancing cell adhesion, but decelerating cell migration and invasion. Furthermore, the knockdown of S100A11 gene expression dramatically induces the cellular response of gastric cancer cells to the first-line chemotherapeutic drugs fluoropyrimidine 5-fluorouracil (5-FU) and cisplatin.

**Conclusion:**

The present study identifies S100A11 as a tumour promoter in gastric cancer. More importantly, the S100A11-specific targeting potentially presents dual therapeutic benefits by not only controlling tumour progression but also sensitising chemotherapeutic cytotoxic response.

**Supplementary Information:**

The online version contains supplementary material available at 10.1186/s12935-021-01949-1.

## Background

Gastric cancer (GC) is ranked as the fifth of the most common malignant tumours and the third leading cause of cancer-related death in the world [1, 2]. Because most patients lack specific symptoms in the early stage of GC, they are dominantly diagnosed with an advanced tumour that presents with metastasis, often missing the optimal operation time and possessing poor prognosis outcome. Accumulating data show that the median survival for patients with metastatic gastric cancer ranges from only 3 to 5 months [3–5]. To date, palliative systemic therapy is the optimal choice for advanced gastric tumours, but the initial response to drugs is different among distinct patients. Moreover, chemoresistance is one of the main clinical challenges, which limits the efficiency of chemotherapy and often leads to treatment failure. Presently, most of the chemotherapy regimens usually induce DNA damage and trigger apoptosis leading to cell death [6]. However, tumour cells could increase DNA repair ability and deregulate apoptosis signaling pathways to resist the therapy response induced by the drugs. Therefore, enhancing apoptosis is an important approach to sensitise tumour response to chemotherapies and control tumour progression. Some molecules have been described to coordinate with drugs and positively regulate cell apoptosis upon chemotherapy, which may affect tumour resistance and improve the therapeutic efficiency to combat cancer.

S100A11, also named calgazzarin, is a member of the S100 family which belongs to calcium-binding proteins with two EF-hands [7]. S100A11 protein is a small molecular weight (13 kDa) acidic protein and shows altered expression in various solid tumours, including lung cancer [8], colorectal cancer [9], pancreatic cancer [10] and prostate cancer [11]. S100A11 protein plays a broad role in intracellular and extracellular biological functions, such as cell differentiation, cell cycle, cell apoptosis, cell proliferation, cell migration and invasion [12, 13]. Additionally, the S100 family has been demonstrated to have a link with cancer stem cells, which play crucial roles in maintaining self-renewal or cancer stem-like properties [14]. An increasing number of papers have reported that S100A11 behaves as a tumour promoter in prostate, pancreatic, breast, ovarian and colorectal carcinoma, but inhibits tumour progression in bladder cancer [9, 11, 15–18]. The translocation of S100A11 from the cytoplasm to the nucleus, induced by DNA damage, could increase the protein level of p21 and regulate the cell cycle [19]. S100A11 knockdown in non-small cell lung cancer induces a strong potential ability of apoptosis upon treatment with chemotherapy drugs [20]. Furthermore, S100A11 has previously been shown to interact with many target proteins, which lead to cancer initiation and progression, such as RAGE, Annexin family, p21/WAFI, p53 and other S100members [21–25].

In the present study, we demonstrate that S100A11 displays higher expression in GC tissues compared to adjacent normal tissues and leads to poor survival in GC patients. S100A11 promotes GC cell migration and invasion via inducing EMT and MMPs. Furthermore, we show for the first time that knockdown of S100A11 in GC cell lines results in the strong induction of apoptosis upon treatment with cisplatin or 5-fluorouracil, suggesting that S100A11 represents promising therapeutic targets to combat GC, or performs as a potential molecular marker to predict the effectiveness of chemotherapy of GC.

## Materials and method

### Cell lines and cell culture

AGS and HGC27 human gastric cancer cell lines were obtained from the European Collection of Animal Cell Cultures (ECACC, Salisbury, UK). The cells were cultured with Dulbecco’s modified Eagle’s medium (DMEM)-F12 medium containing 10% fetal calf serum (FCS) and antibiotics which was named as a normal medium, and maintained in a 5% CO_2_ incubator at 37 °C.

### Patients and human GC tissue

A total of 324 GC specimens along with matched adjacent normal gastric tissues from GC patients, who were diagnosed and surgically treated at Peking University Cancer Hospital over a period from 2004 to 2007 with follow-up information up to 2012, were included in this study. In addition, another 87 pairs of GC and corresponding adjacent normal tissues collected from GC patients, who had been treated with perioperative chemotherapy between 2006 and 2007, were analyzed to detect the relationship between S100A11 and chemotherapy resistance. All the tissues were stored immediately after surgery at − 80 °C or subjected to RNA isolation. Clinicopathological factors were recorded and stored in the patient database. GC stage was classified according to the 2010 tumor-node-metastasis (TNM) classification recommended by the American Joint Committee on Cancer (AJCC 7th edition). All patients signed an informed consent form, and the study was approved by the Ethics Committee of Peking University Beijing Cancer Hospital.

### Lentiviral vector transduction of GC cells for stable S100A11 knockdown

Lentivirus plasmids containing a short hairpin RNA (shRNA)for S100A11and its negative control were purchased from Vector Builder (Vector Builder, Cyagen Biosciences, Santa Clara, USA). Lentivirus was produced by the co-transfection of 293 T cells with a pLenti vector (pLV-shControl or pLV- shS100A11) and lentiviral packaging mix (psPAX2 packing plasmid and pMD2.G envelope plasmid), according to the manufacturer’s instruction. Lentivirus-containing supernatant was harvested at 24 and 48 h post-transfection, filtered and stored at − 80 °C. Briefly, viral particles were added into AGS and HGC27 cells with 8 µg/ml Polybrene for 24 h, and then the fresh medium was changed. After incubation for another 24 h, the infected cells were subjected to a selection medium with 2 μg/ml puromycin. Finally, stable silencing S100A11GC cells were maintained in a medium with 0.6 μg/ml puromycin.

### RNA isolation, reverse transcriptional PCR, conventional polymerase chain reaction (PCR)

Total RNA was isolated separately from GC cells and frozen tissues using TRI reagent (Sigma-Aldrich), and one-step reverse transcribed into cDNA using Promega Reverse Transcription kit (Promega, Dorset, UK) according to the manufacturer’s instructions. PCR was performed on SimpliAmp Thermal Cycler (Applied Biosystems, Paisley, UK) using GoTaq Green MasterMix (Promega, Dorset, UK) in a final volume of 16 μl. GAPDH was used as an endogenous control for each sample and the PCR products were visualized using 1.5% agarose gels stained with SYBR Safe (Invitrogen).

### Transcript quantification of S100A11 in GC cells and GC tissues by quantitative real-time PCR (qPCR)

S100A11expression in GC cells and tissues was detected by qPCR, which was run on the StepOne Plus Real-Time PCR System (Applied Biosystems, Paisley, UK), and performed with a Precision FAST 2X qPCR MasterMix (Primer Design Ltd., Chandler’s Ford, UK) in a final volume of 10 µl, using GAPDH or β-actin as an internal control. Reaction conditions were as follows: 94 °C for 10 min, followed by 100 cycles of 94 °C or 10 s, 55 °C for 30 s and 72 °C for 10 s. The primer sequences used for PCR and qPCR are listed in Additional file 4: Table S1.

### Western blot analysis

Cells were washed by ice-cold PBS twice, and extracted by RIPA buffer with protease inhibitor and phosphatase inhibitor. Protein concentration was qualified by DCTM protein assay kit (Bio-Rad, Laboratories, Hemel-Hempstead, UK). Thirty μg of each protein sample was transferred onto a 0.45 μm polyvinylidenedifluoride (PVDF) membrane (Millipore, Billerica, MA) after separation by 10 or 12% sodium dodecyl sulfate–polyacrylamide gel electrophoresis (SDS-PAGE). The membrane was blocked with 5% nonfat milk for 1 h at room temperature, then incubated in primary antibody (1:500) at 4 °C overnight and secondary antibody (1:2000) for 1 h. The primary antibodies were S100A11, cleaved PARP (detecting both full-length and cleaved PARP) (eBioscience Inc., San Diego, CA, USA); p21WAF1, p53 (DO-1) and GAPDH (Santa Cruz Biotechnology, Inc., Santa Cruz, USA). The results shown are representative of three independent experiments.

### Conditioned medium

Cells were cultured in T25 flasks with a normal medium. At 70–80% confluence, cells were washed twice with PBS and continued to incubate in a serum-free medium. After 48 h, the conditioned medium was collected and concentrated by Amicon® Ultra-4 Centrifugal Filter Devices (50 kDa, Sigma-Aldrich) before use or stored at -80 °C.

### Treatment with Cisplatin and 5-fluorouracil (5-FU)

When reaching 70–80% confluence, cells were changed into serum-free medium and cultured for 4–6 h, then treated with chemotherapeutic drugs as follows: 20 μM cisplatin and 38 μM 5-fluorouracil for 24 h, followed by Western Blot and FACS analysis. For caspase-3/7 activity investigation, cells were treated with 6.25 μM cisplatin and 19 μM 5-fluorouracil for 24 h.

### Colony assay

Transfected GC cells were seeded into 6-well plates at 400 cells/ well and treated with 2 μM Cisplatin or 19 μM 5-fluorouracil for 24 h. After the treatment, the growth medium was replaced with a fresh medium without drugs, and cells were cultured for two weeks. Colonies derived from cells were subsequently fixed in 4% formalin for 30 min and stained with 0.5% (w/v) crystal violet (Sigma-Aldrich, St. Louis, MO, USA) for 15 min. The excessive crystal violet was removed using double-distilled H_2_O. After the stained colonies were photographed, they were completely dissociated in 10% (v/v) acetic acid. The densities of the stained and dissolved colonies were then qualified by measuring the absorbance at 540 nm using a spectrophotometer (Elx800; Bio-Tek, Bedfordshire, UK).

### Cell matrix adhesion assay

Ten micrograms of Matrigel® Basement Membrane Matrix (Corning Incorporated, Flintshire, UK) were used to coat each well of a black 96-well plate. A total of 2 × 104 cells were seeded into each well and incubated for 1 h. Then cells were washed twice by PBS and stained by dissociation solution (CDS; Sigma-Aldrich)/calcein-AM Viability Dye (eBioscience Inc., San Diego, CA, USA) at a ratio of 1.2 μl calcein-AM in 1 ml CDS (100 μl/well) for 30 min. The suspension was read on a GLOMAX® MULTI Detection System (Promega, Dorset, UK) at 495 nm excitation and 519 nm emission and images of the cells were taken using 4 × EVOS FL Auto (Life Technologies). The results were expressed as a fold change of the adherent cell number compared to the lowest value.

### Cell invasion assay

An 8 μm-pore ThinCert™ 24-well plate insert (Greiner Bio-One, Gloucester, UK), previously coated with 100 μg of Matrigel® Basement Membrane Matrix (Corning Incorporated, Flintshire, UK), was used for evaluating the cell invasion. Following this, 5 × 10^4^ cells were seeded in the upper chamber in serum-free medium, and the bottom chamber was filled with DMEM containing 10% FCS. After 24 h of incubation, the chamber was washed gently with PBS and incubated for one hour in 350 μl of dissociation solution/calcein-AM Viability Dye following the method described above. Then the cell suspension was aliquoted into a black 96-well plate and read on a GLOMA® MULTI Detection System and images were taken of the invasive cell groups using 4 × EVOS FL Auto (Life Technologies).

### Cell migration assay

Cell migration was assessed with a wound-healing assay. Cells were seeded into a 24-well plate at a density of 6 × 10^5^ per well and then scratched to create a wound with a pipette tip after cells had formed a monolayer. The wound width was detected using a microscope at 0, 2, 4, 6, 8 and 10 h, and migration distances were measured using Image J software (www.ImageJ.net).

In addition, an electric cell-substrate impedance sensing (ECIS)-Zθ instrument (Applied Biophysics Ltd.; Troy, NJ, USA) was used to monitor cell migration, as described previously [26]. Briefly, 6 × 10^4^ cells, diluted in DMEM with 10% FCS, were seeded into each well of ECIS 96-plate arrays. Wounding was carried out by applying electric current (3000 μA, 60 kHz) once a confluent monolayer had formed. The migration data were collected continuously for 12 h.

### Measurement of caspase 3/7 activity

Cells were transfected as above and loaded into a white wall 96-well plate at a density of 5 ˣ 10^3^ per well. After being starved for 4–6 h in serum-free DMEM, cells were treated with Cisplatin and 5-FU at the indicated concentration for 24 h, while DMSO or PBS was used as a control. Then the levels of caspase-3/7 activity were assessed using a Caspase-Glo® 3/7 Assay (Promega) according to the manufacturer’s instructions. The luminescence of each sample was read on a GLOMA® MULTI Detection System.

### Kinexus™ protein microarray

The stable gastric cancer cell lines containing S100A11-kd or Scr were cultured in T75 flasks until reaching approximately 80% confluence followed by 2% FCS medium for 24 h before protein extraction. Cells were washed by iced-PBS twice and lysed in a lysis buffer containing 100 mM tris, 10% 2-mercaptoethanol, 1% NP-40, 50 mM NaFI, 2 mM 4-(2-aminoethyl) benzenesulfonyl fluoride, 14 μM E-64, 130 μM bestatin, 1 μM leupeptin, 0.3 μM aprotinin and 1 mM EDTA. The cell lysates were delivered for the Kinexus™ antibody microarray analysis (Kinexus Bioinformatics, Vancouver, British Columbia, Canada) in dry ice to determine global signalling events.

### Flow cytometric analysis

For protein expression analysis, cells were fixed with IC Fixation Buffer (eBioscience Inc. San Diego, USA) in the dark at room temperature for 30 min, and then incubated in ice-cold 100% methanol at 4 °C for 30 min. After blocking in 1% BSA in PBS, cells were incubated in PE-conjugated cleaved PARP1 antibody (1:40, eBioscience Inc. San Diego, USA) overnight at 4 °C. In parallel, a mouse PE-conjugated IgG2b K Isotype antibody (1:10,000, eBioscience Inc. San Diego, USA) was used as a control. The next day, samples were washed by PBS twice and detected by using a BD CANTO II flow cytometer (BD Bioscience).

### Hierarchical Clustering on Principal Components (HCPC) multivariate analysis

An unsupervised approach of hierarchical clustering and principal component analysis was performed using the R programming language version 4.0.3 (https://www.r-project.org) with packages of FactoMineR [27] and Factoextra [28]. The transcript expression data of all the S100 family members in gastric cancer were extracted from TCGA.

### Statistical analysis

All statistical analyses were performed using R programming language or GraphPad Prism 6.0 (GraphPad Software, La Jolla, CA, USA). The results were expressed as mean ± standard deviation (SD) of three independent experiments. The overall survival curve was drawn by the Kaplan–Meier plots and a log-rank test was used to estimate the difference between groups. Statistical evaluation was performed using Student’s t-test if data followed a normal distribution or Mann–Whitney U-test if data did not follow a normal distribution. A p-value less than 0.05 was considered statistically significant.

## Results

### Clinical relationship of the S100A11 transcript and GC

We first determined the gene expression level of S100A11 by qPCR in GC tissues collected from an in-house cohort of patients. As shown in Table 1, S100A11 expression exhibited significantly higher in GC tissues than adjacent normal tissues (P < 0.0001). However, we did not observe any statistical significance from GC groups with advanced tumour infiltration (T3 + T4) or TNM stages (TNM III + IV).

**Table 1:** The relationship between S100A11 transcript expression and the clinicopathological features in gastric cancer. Mann-Whitney U-test was used to compare the difference between two groups

	N	Median	P
Tissue			
Tumor	237^**§**^	580	** < 0.0001**
Normal	292^**§**^	0	
Sex			
Male	175	621	0.8394
Female	62	368	
Depth of invasion			
T1 + T2	25	735	0.6443
T3 + T4	205	567	
Lymph node status			
N0	48	452	0.4236
N1 + 2 + 3	185	589	
Distance metastasis			
M0	200	574	0.167
M1	36	572	
TNM staging			
TNM1 + 2	54	452	0.5954
TNM3 + 4	175	589	
Differentiation			
Diff-H	1	232.84	
Diff-HM	5	3531	
Diff-M	46	475	0.0901
Diff-ML	55	947	0.1726
Diff-L	101	327	0.0565
Clinical outcome			
Alive	92	742	0.5648
Died	144	384	

Bold value indicate P value less than 0.05 was considered statistically significant

^§^The missing clinical samples were excluded

**Fig. 1:**
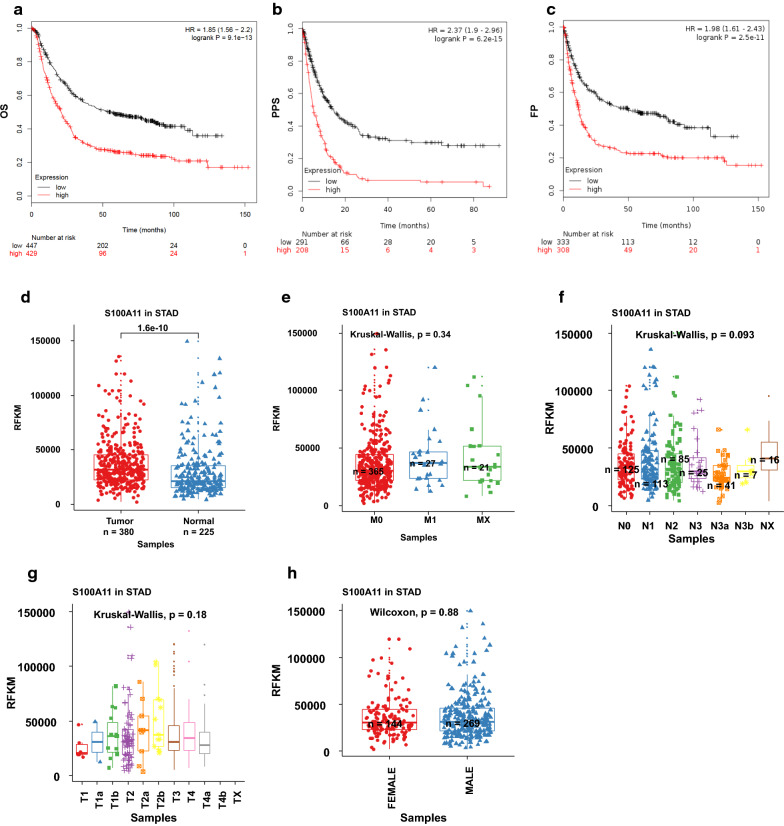
Association of S100A11 gene expression with the survival and clinical features of gastric cancer patients. The survival analysis of S100A11 (probe_ID: 200660_at) was conducted on the KMPlot (https://kmplot.com). **a** Overall survival (OS). **b** Post progression survival (PPS). **c** First progress survival (FP). The association of S100A11 and the clinicopathological features of GC was analysed using the TCGA data. **d** Tumour vs normal. **e** Metastasis status. M0: no metastasis; M1: with metastasis. Mx: unknown metastasis status. **f** Lymph node metastasis stages. **g** Tumour stages. **h** Gender. The difference between the two groups was analysed using the Wilcoxon test, while the Kruskal–Wallis test was used to compare the difference among multiple groups

We then conducted the survival analysis of S100A11 in GC using the KMplot dataset (https://kmplot.com/analysis). As shown in Fig. 1a-c, the gene expression of S100A11 was negatively associated with overall survival (OS) (HR = 1.85 (1.56–2.20), logrank P < 0.0001), post-progression survival (PPS) (HR = 2.37 (1.9–2.96), logrank P < 0.0001) and first progression survival (FP) (HR = 1.98 (1.61–2.43), logrank P < 0.0001). The differential expression analysis of the TCGA stomach adenocarcinoma (STAD) data (https://www.cancer.gov/tcga) indicated that the gene expression level of S1000A11 was higher in the tumour tissues than in the normal ones (p < 0.0001. Figure 1d). There was no significance observed among other clinicopathological features including M status, T stages, and sex (Fig. 1e-h).

### Expression of S100A11 protein in GC tissues

**Fig. 2:**
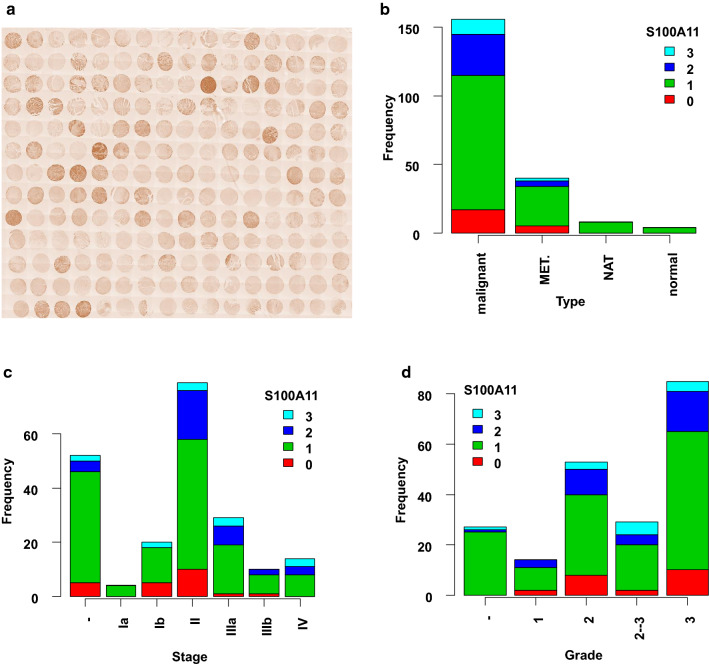
Immunohistostaining of S100A11 protein in gastric cancer tissue microarray. **a** The thumbnail image of the immunohistostaining of the GC tissues using a specific S100A11 antibody. The staining intensity was categorised into 4 levels: 0 negative; 1 weak; 2 intermediate; 3 strong. **b** Stacked frequency plot of the tumour types. MET, metastasis; NAT, normal adjacent tissue; **c** Stacked frequency plot of the tumour stages. **d** Stacked frequency plot of the tumour grades

We then evaluated the expression of the S100A11 protein in GC tissues by immunohistochemistry. The thumbnail image of the tissue array after staining with the S100A11 is shown in Fig. 2a. It showed that the high intensity (2 and 3) of the S100A11 staining occurred more frequently in malignant and metastatic (MET) tumour tissues than in the normal adjacent (NAT) and normal stomach tissues (Fig. 2b). Further, a higher percentage of the high intensity of the S100A11 appeared in later stages (Stage >  = 2) of the tissues (Fig. 2c). The high intensity of the S100A11 staining was observed more in the higher grade tumour tissue as well (Fig. 2d). Taken together, the immunohistochemistry data demonstrate that S100A11 protein is expressed at higher levels in tumour tissue with stage and grade dependence.

### Effect of S100A11 on epithelial-mesenchymal transition (EMT) and matrix metalloproteinase (MMPs) molecules in GC cells

**Fig. 3:**
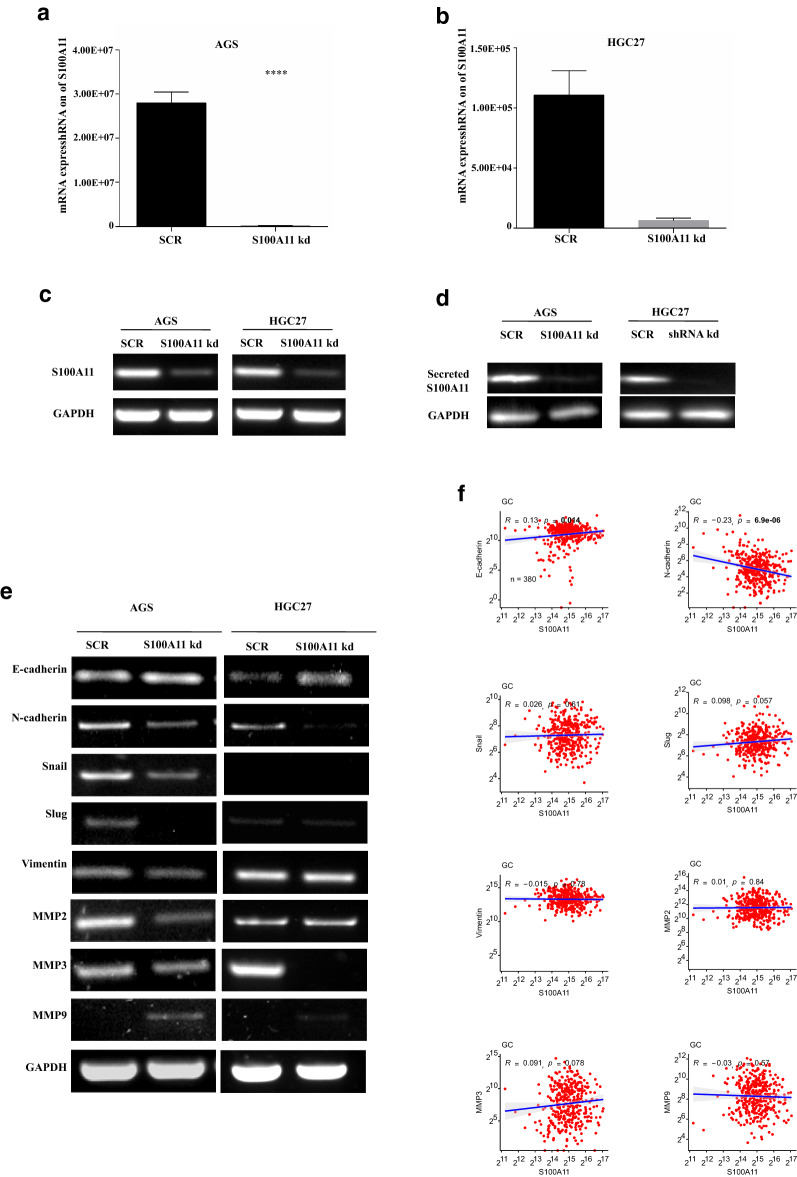
Effect of the knockdown of S100A11 on the regulation of EMT and MMP markers. The stable gastric cancer cells following the S100A11 knockdown were validated using qRT-PCR in AGS cells (**a**), HGC27 cells (**b**) and Western blotting (**c**). **d** The reduction of the secreted S100A11 protein in the culture medium indicated by Western blotting using the cellular lysate as a control. **e** Quantification of EMT and MMP markers in cells by semi-quantitative RT-PCR. **f** The correlation analysis of the transcript expression levels of S100A11 with EMT and MMP genes using the TCGA-STAD dataset

To elucidate the function of S100A11, we knocked down this gene in gastric cancer cells and validated the knockdown efficiency by qPCR and Western blotting (Fig. 3a–c). Interestingly, we noticed that S100A11 can be secreted into the conditioned medium and the expression of secreted S100A11 was decreased in S100A11-silencing cells (Fig. 3d).

EMT is a process in which epithelial cells acquire mesenchymal, fibroblast-like properties and show reduced intercellular adhesion, critical for invasive and metastatic progression in cancer. Here, we found that S100A11 depletion resulted in an upregulation of E-cadherin, and downregulation of N-cadherin, Snail and Slug molecules in both cell lines, whilst Vimentin decreased only in AGS cells with S100A11-kd. Moreover, we detected MMP expression and noticed that MMP2 and MMP3 were depressed in both cell lines, while MMP9 slightly increased in the S100A11-kd AGS cells compared to the control (Fig. 3e). Therefore, S100A11 may promote invasion and migration of GC cells by inducing EMT and MMP production.

To understand whether there is an intrinsic relationship of S100A11 with the EMT and MMP markers, we conducted the correlation analysis using the TCGA STAD RNA-Seq dataset, as shown in Fig. 3f, in GE tissues. S100A11 was positively correlated with E-cadherin (R = 0.13, p < 0.05), while negatively correlated with N-cadherin (R = − 0.23, p < 0.0001). There was no significant correlation between S100A11 and other markers.

### Knockdown of S100A11 enhances cell adhesion but decelerates the migration and invasion of gastric cancer cells

**Fig. 4:**
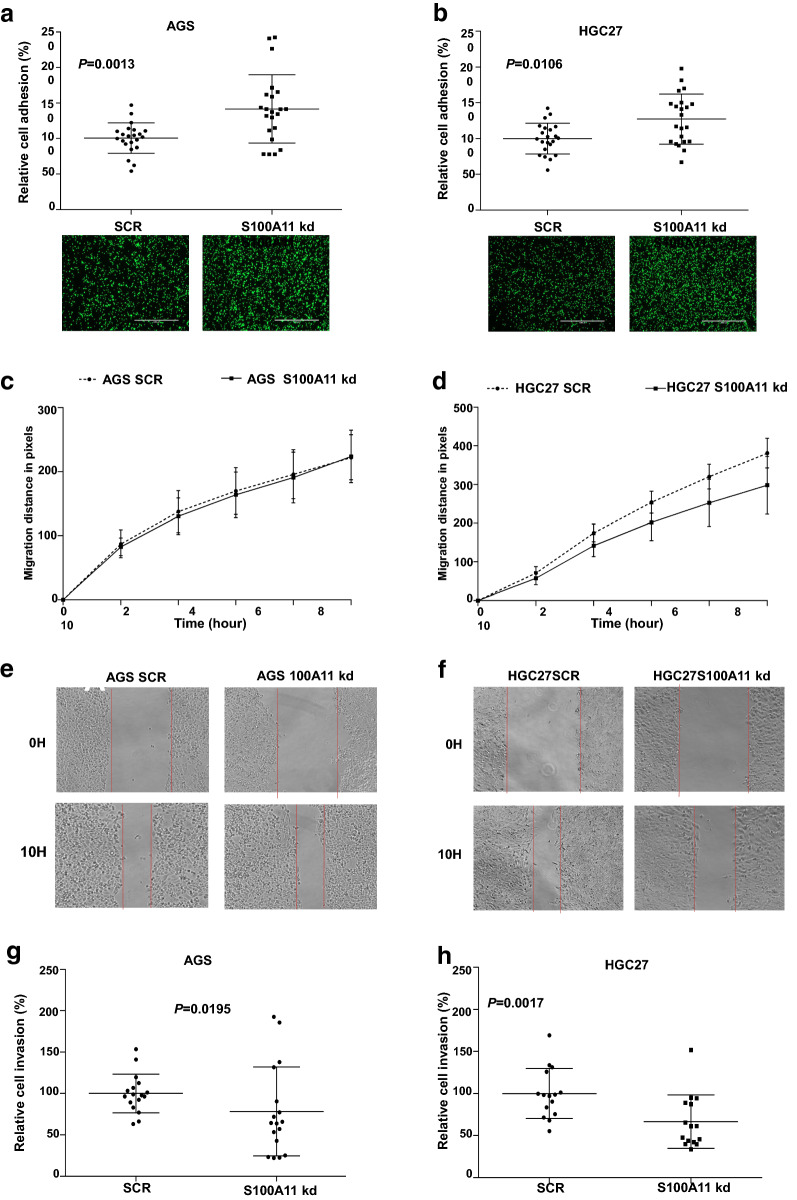
Effect of the S100A11 knockdown on adhesion, migration and invasion of gastric cancer cell lines. **a** Adhesion of the AGS cell lines with representative images beneath. **b** Adhesion of the HGC27 cell lines. **c** The migration analysis of the AGS cell lines using the wound healing assay. **d** The migration analysis of the HGC27 cell lines using the wound healing assay. **e** Representative images of the stable AGS cell lines during the wound healing assay. The red lines highlight the wound gap. **f** Representative images of the stable HGC27 cell lines during the wound healing assay. **g** Invasion analysis of the AGS cell lines. **h** Invasion analysis of the HGC27 cell lines

Next, we performed a series of functional assays in AGS and HGC27 cells to determine the role of S100A11 in gastric tumourigenesis. In vitro cell–matrix adhesion was used to detect the effect of S100A11 knockdown on the adhesive ability of GC cells to the extracellular matrix. A significant increase of cell–matrix adhesion was observed in AGS and HGC27 GC cells after S100A11 knockdown (P = 0.0013 and P = 0.0106, respectively, Fig. 4a, b). Furthermore, the knockdown of S100A11 suppressed the migration in HGC27 cells compared to controls, but there was no difference in AGS cells (Fig. 4c–f). Besides, the knockdown of S100A11 in both AGS and HGC27 cells reduced the cellular invasive ability compared to their respective controls (P = 0.0195 and P = 0.0017, respectively, Fig. 4g, h). The data, therefore, demonstrated that S100A11 plays a role in promoting migration and invasion in GC cells.

### Knockdown of S100A11 attenuates chemotherapy resistance in GC cells

**Fig. 5:**
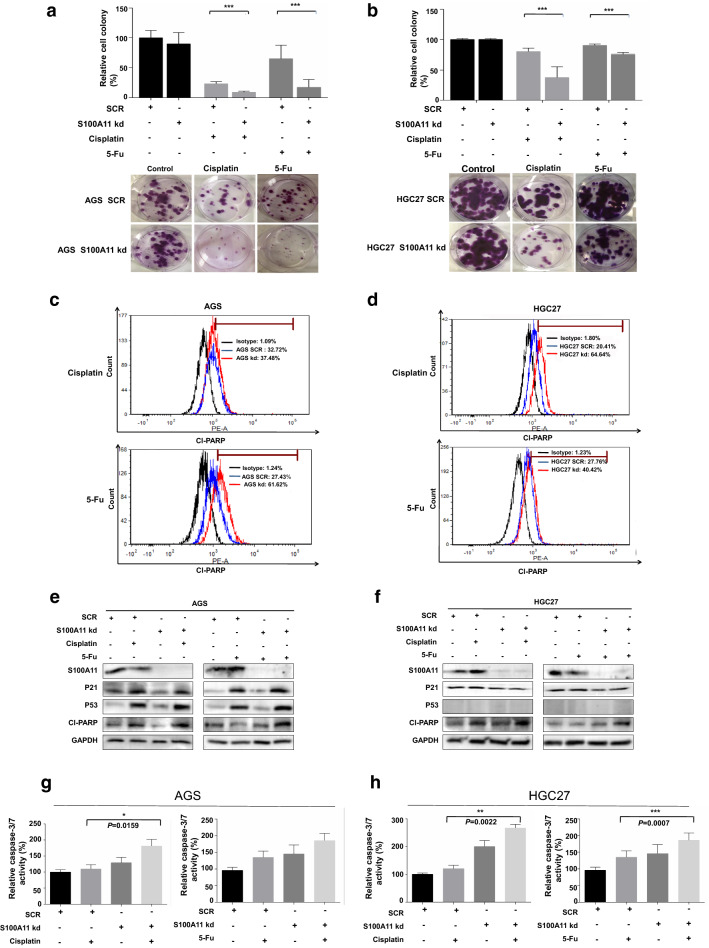
Role of S100A11 in the cellular response to therapeutic drugs. Effects of the S100A11 knockdown on colony formation in response to cisplatin and 5-Fu were evaluated in the stable AGS (**a**) and HGC27 (**b**), respectively. The representative images of colony formation are shown at the bottom. The change of the apoptosis levels indicated by FACS-based cleaved PARP in the stable AGS (**c**) and HGC27 cells (**d**), respectively. Western blotting was used to quantify the change of P53, P21 and cleaved-PARP proteins in the AGS (**e**) and HGC27 cells (**f**), respectively. Caspase 3/7 assay was used to verify the apoptosis in response to cisplatin or 5-Fu in the stable AGS (**g**) and HGC27 cells (**h**), respectively

We then investigated whether S100A11 knockdown contributed to chemotherapy resistance. We performed a cell colony array to evaluate the effect of S100A11 on cellular response to cisplatin and 5-FU resistance. As shown in Fig. 5a, b, there was a significant decrease in cell colony formation in the S100A11-knockdown groups. Such decreases in drug resistance were accompanied by the significant increase of cell apoptosis in the S100A11-kd AGS and S100A11-kd HGC27 cells after the treatment with cisplatin or 5-FU for 24 h, as indicated by the increased level of cleaved PARP via FACS and Western blot (Fig. 5c–f). Moreover, S100A11 downregulation resulted in a significant increase of caspase-3/7 activity in GC cells after treatment with cisplatin and 5-FU (Fig. 5g, h). We also noticed that S100A11 knockdown induced p21 and p53 protein expression in AGS cells compared to the control cells (Fig. 5e). Collectively, our data suggest that silencing S100A11 in GC cells may decrease the resistance to cisplatin or 5-FU resistance by inducing apoptosis.

### S100A11 participates in multiple signalling pathways as indicated by high through antibody array

**Fig. 6:**
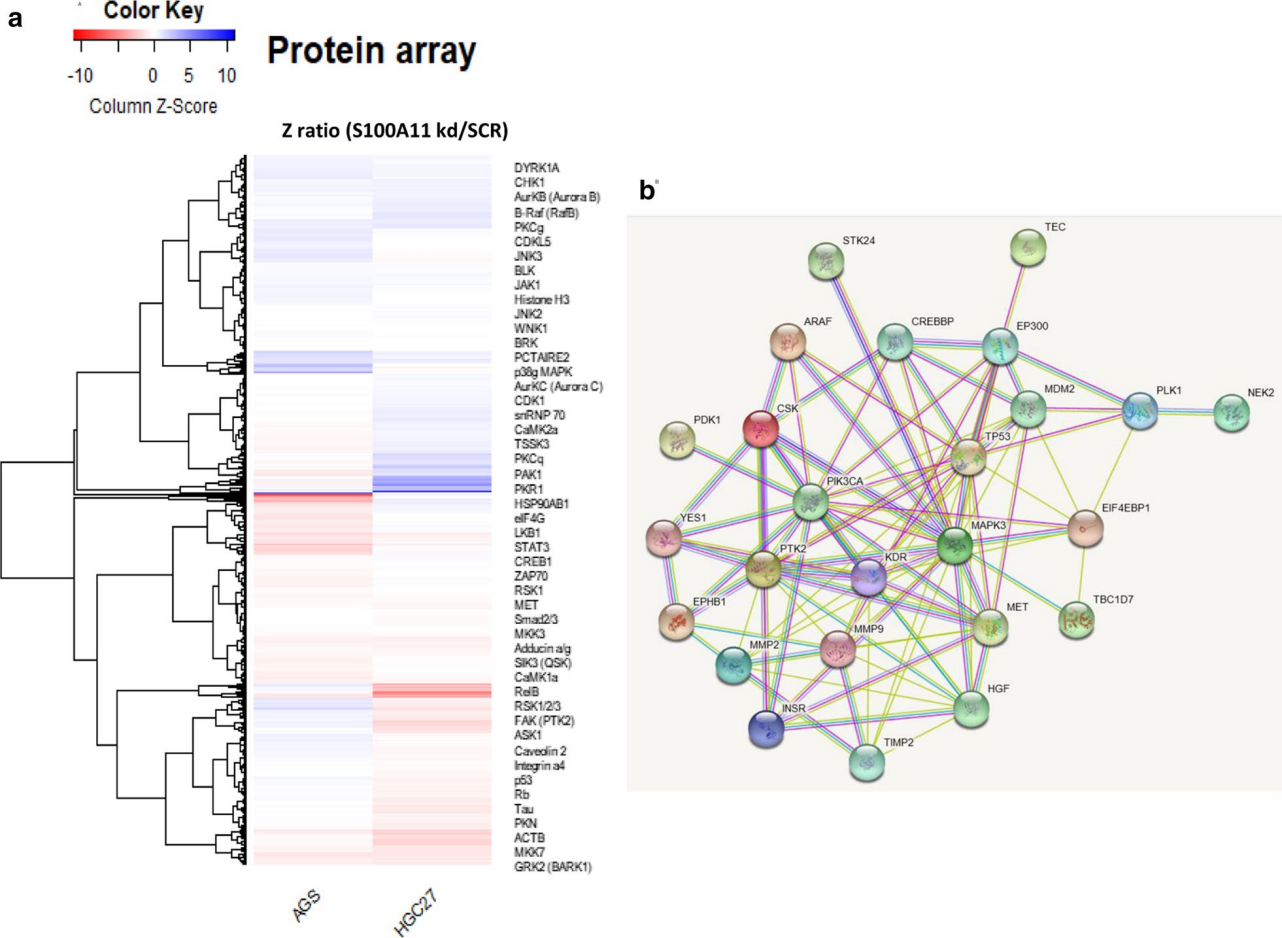
The comparison of the key signalling transduction checkpoints by high throughput antibody array. **a** The heatmap plot showing the alteration of the signalling proteins after the S100A11 knockdown in the stable AGS and HGC27 cells. **b** The altered Protein–protein interaction network as a consequence of S100A11 knockdown was identified by enrichment analysis.

To determine the role of S100A11 in regulating cell signalling in GC cells globally, we conducted high throughput Kinex antibody array using the stable cell lines following S100A11 knockdown. The regulated proteins, in response to the S100A11 knockdown in two parental cell lines, were compared using heatmap plotting (Fig. 6a). We further performed the signalling pathway enrichment analysis and observed that S100A11 is highly involved in the regulation of multiple signally pathways including MAPK3 (ERK1), PIK3CA, HGF/MET, CREBBP and MMP9 (Fig. 6b). The thumbnail images of the antibody array are shown in Additional file 1: Figure S1.

### The expression profile of S100A11 is dimensionally close to S100A10 and S100A6

The HCPC analysis indicated that S100A11 is clustered as a distinctive factor that is dimensionally distant to most of the other S100 family members in GC. S100A11 contributes predominantly to the first principal component (82.5%) while was distant from the second one (7.9%). Dimensionally, S100A11 is close to S100A6 and S100A10, as shown by both the PCA factor map and hierarchical clustering dendrogram, although S100A6 was marginally more similar to S100A11 compared to S100A10 (Additional file 2: Figure S2).

## Discussion

In this study, we find that the overexpression of S100A11 is associated with poor survival in GC patients as indicated by Kaplan–Meier analysis. S100A11 transcript levels are upregulated in clinical GC samples compared with normal tissues. Additionally, S100A11 protein level tends to be increased in advanced stages of GC or deeper tumour infiltration. S100A11 is a member of the S100 protein family which is composed of 21 members which have structural similarity but are functionally noninterchangeable. The S100 family members have been considered as both intracellular calcium sensors and extracellular factors which regulate multiple cellular processes depending on its stimuli and effectors [29]. It is known that the S100A11 gene is located at 1q21.3, a site that is enriched with another seventeen S100 gene members. The dysregulation of S100A11 is linked with oncogenic activities including cancer progression. It has been observed to be overexpressed in many human solid tumours, such as pancreatic, melanoma, breast, ovarian and colorectal cancer [30–34]. Here we propose S100A11 may play a positive role during the development and progression of GC.

Previous studies suggest that S100A11 plays a role in a variety of physiological processes in tumours which include regulation of cell differentiation, invasion, migration cell cycle and apoptosis [29]. We show herein that the knockdown of S100A11 results in diminished invasion and migration capabilities of GC cells with the upregulation of epithelial markers and downregulation of mesenchymal markers. Thus, S100A11 may contribute to metastasis and poor prognosis in GC by promoting EMT. EMT is a biological programme that allows a polarized epithelial cell to undergo multiple changes that enable it to acquire a mesenchymal cell phenotype [35]. The acquisition of EMT features is crucial for carcinogenesis, invasion and metastasis. EMT is also a known risk factor that contributes to cancer recurrence and poor survival among patients with various solid cancers including breast [39], bladder [40], gastric [41], and colon cancer [42].

We demonstrate that the knockdown of S100A11 upregulates the expression of E-cadherin. However, the correlation analysis using the TCGA data indicates that S100A11 positively correlates with E-cadherin. Unlike E-cadherin, N-cadherin is considered as a mesenchymal marker of EMT [39]. We show that the knockdown of S100A11 downregulates the expression of N-cadherin, while S100A11 negatively correlates with N-cadherin indicated by the analysis of TCGA data. This suggests that S100A11 may regulate the expression of E-cadherin/N-cadherin at the protein level but not the transcript level. The downregulation of E-cad is a critical step in the EMT process [36], and E-cadherin is considered to be a tumour suppressor in gastric cancer [37, 38]. There has been no previous study on the role of S100A11 in the regulation of E-cadherin/N-cadherin in gastric cancer. However, a study on cervical cancer also suggests that S100A11 upregulates N-cadherin while downregulates E-cadherin [40], implying that this may be common in several solid cancers.

In addition, we find that S100A11 downregulates MMP9 but upregulates MMP2 and MMP3. The human MMP protein family has 23 members and participates in cancer cell invasion and metastasis in diverse ways. Accumulating reports show that overexpression of certain MMPs (e.g. MMP2, MMP3 and MMP9) is associated with EMT during cancer initiation and progression [41]. We, therefore, propose that S100A11 may modulate the EMT programme by regulating the profiles of certain MMPs.

Here, we reveal that the knockdown of S100A11 leads to lower resistance to cisplatin or 5-FU-induced apoptosis in companies with an enhanced cleaved-PARP level in GC cells, and reduced rates of proliferation. Clinical data suggest that patients who positively respond to preoperative chemotherapy have a significantly longer survival time than non-responders [42]. However, tumour chemoresistance remains one of the most significant challenges to the successful treatment of GC [43]. Here our data unveil a new insight into the molecular mechanism whereby S100A11 plays a role in chemoresistance in GC.

The HCPC analysis indicates that S100A11 is indeed unique and distinctive when all of the 21 S100 family members are hierarchically clustered in GC. S100A11 contributes predominantly to the first principal component and keeps its centroid distance from most of the other members. Within two relatively close S100 neighbours, high gene levels of both S100A6 and S100A10 are associated with poor survival of patients with GC [44, 45]. They may promote malignant properties of gastric cancer cells through modulating multiple signalling pathways such as various annexins and mTOR [45–48]. S100A11 may have the similarity of cellular functions by sharing the interaction of those protein effectors like S100A6 and S100A10 [49, 50].

The present study sheds light on the role of S100A11 in tumor progression and chemoresistance in GC. The underlying mechanisms of this finding require further investigation. However, recent studies from other cancer types provide some possible leads. In hepatocellular carcinoma, S100A11 is involved in inflammation which may contribute to cancer development [51]. Also, as a Ca2 + -binding protein, S100A11 may disrupt the repair of DNA double-strand breaks and genome integrity by interacting with some key DNA strand exchange enzymes such as RAD51 [52]. Further, for translational research on a potential therapy by targeting S100A11, a humanised in vivo study may be vital to be developed for the first instance in order to mimic a *bona fide* GC environment.

## Conclusion

In summary, we reveal the role of S100A11 in GC and the relationship between S100A11 and chemotherapy resistance in human tumours. S100A11 is frequently overexpressed in both human GC tissues and cell lines, and positively correlates with poor survival in GC patients. Reduction of the expression of S100A11 in GC cells inhibits migration and invasion but decreases adhesion in GC cells. We observed that S100A11 functions as a regulator of EMT and MMP proteins. Further, S100A11 knockdown leads to the decreased resistance to cisplatin or 5-FU in GC cells. Taken together, this study suggests that S100A11 may present an independent prognostic factor to predict the effectiveness of preoperative chemotherapy in patients with GC. To the best of our knowledge, this is the first report describing that S100A11-specific targeting potentially presents dual therapeutic benefits by not only controlling tumour progression but also sensitising a chemotherapeutic cytotoxic response (Schematic illustration in Additional file 3: Figure S3).

## Supplementary Information

**Additional file 1**: **Figure S1**. Images of the Kinex high throughput antibody array.

**Additional file 2**: **Figure S2**. Hierarchical Clustering on Principal Components (HCPC) multivariate analysis of the human S100 family member genes in gastric cancer.

**Additional file 3**: **Figure S3**. Schematic illustration of the functions of S100A11 in gastric cancer cells.

**Additional file 4**: **Table S1**. Sequences of primer pairs for RT-PCR.

## Data Availability

All data generated or analyzed during this study are included in this published article and its supplementary information files.

## Abbreviations

GC: Gastric cancer
OS: Overall survival
HR: High Risk
HCPC: Hierarchical clustering and principal component analysis
HR: Hazard ratio
EMT: Epithelial-mesenchymal transition
DMEM: Dulbecco’s modified Eagle’s medium
PPS: Post progression survival
FP: First progress survival
FACS: Fluorescence-activated cell sorting
5-FU: 5-Fluorouracil
PVDF: Polyvinylidenedifluoride
TCGA: The Cancer Genome Atlas
STAD: Stomach Adenocarcinoma
