# Supplementary figures and images for: Dual effects of targeting S100A11 on suppressing cellular metastatic properties and sensitizing drug response in gastric cancer
Source: Cancer Cell Int. 2021 Apr 30;21:243. doi: 10.1186/s12935-021-01949-1 (PMC8086328; doi:10.1186/s12935-021-01949-1)

Sup Figure 1

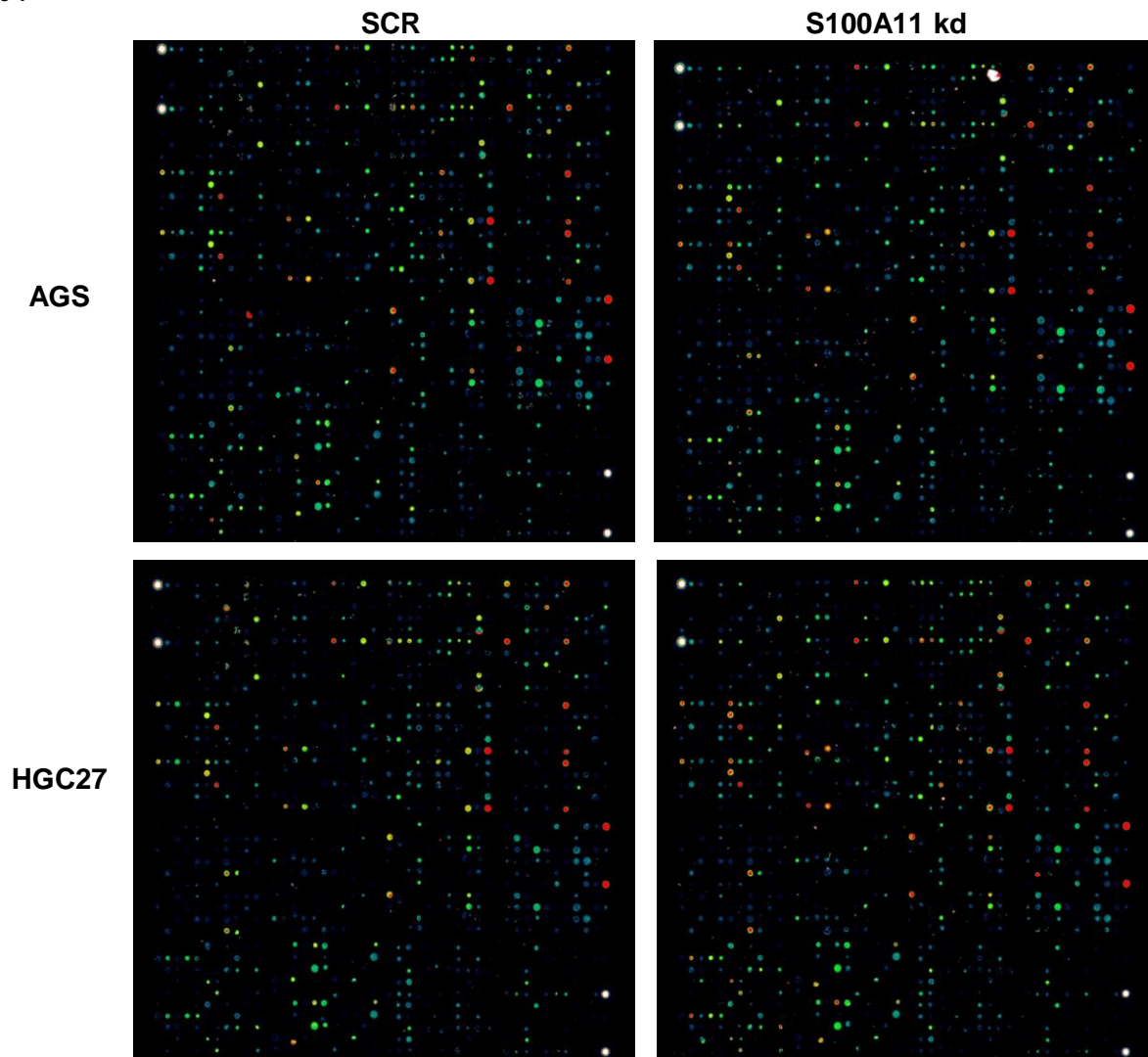

Supplement: Supplementary file 1 — Additional file 1: Figure S1. Images of the Kinex high throughput antibody array. [file 12935_2021_1949_MOESM1_ESM.pdf]

Sup Figure 2

## Factor map

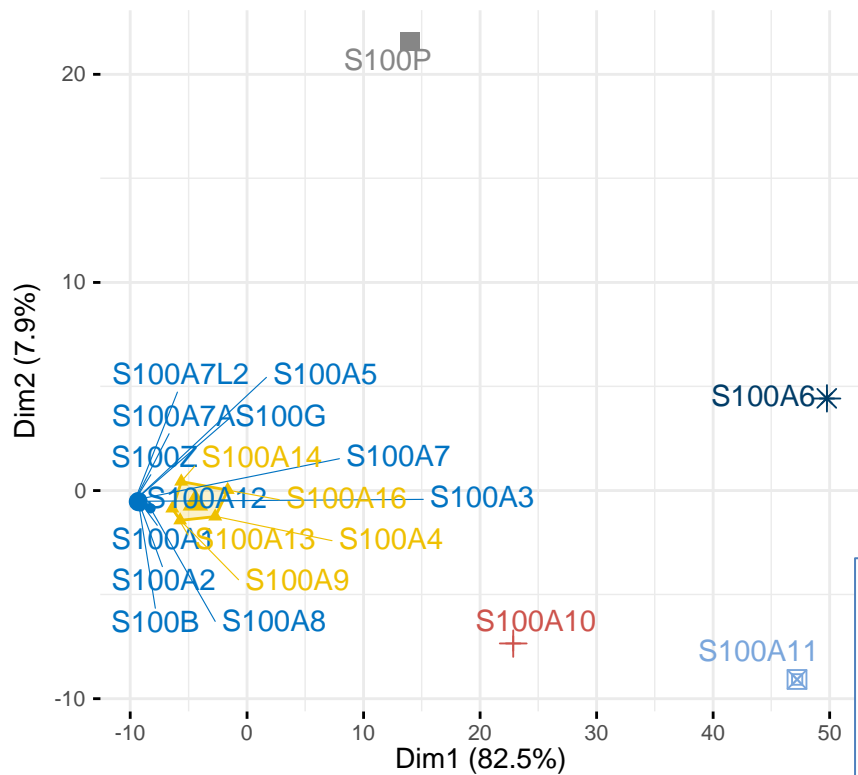

Supplement: Supplementary file 2 — Additional file 2: Figure S2. Hierarchical Clustering on Principal Components (HCPC) multivariate analysis of the human S100 family member genes in gastric cancer. [file 12935_2021_1949_MOESM2_ESM.pdf]

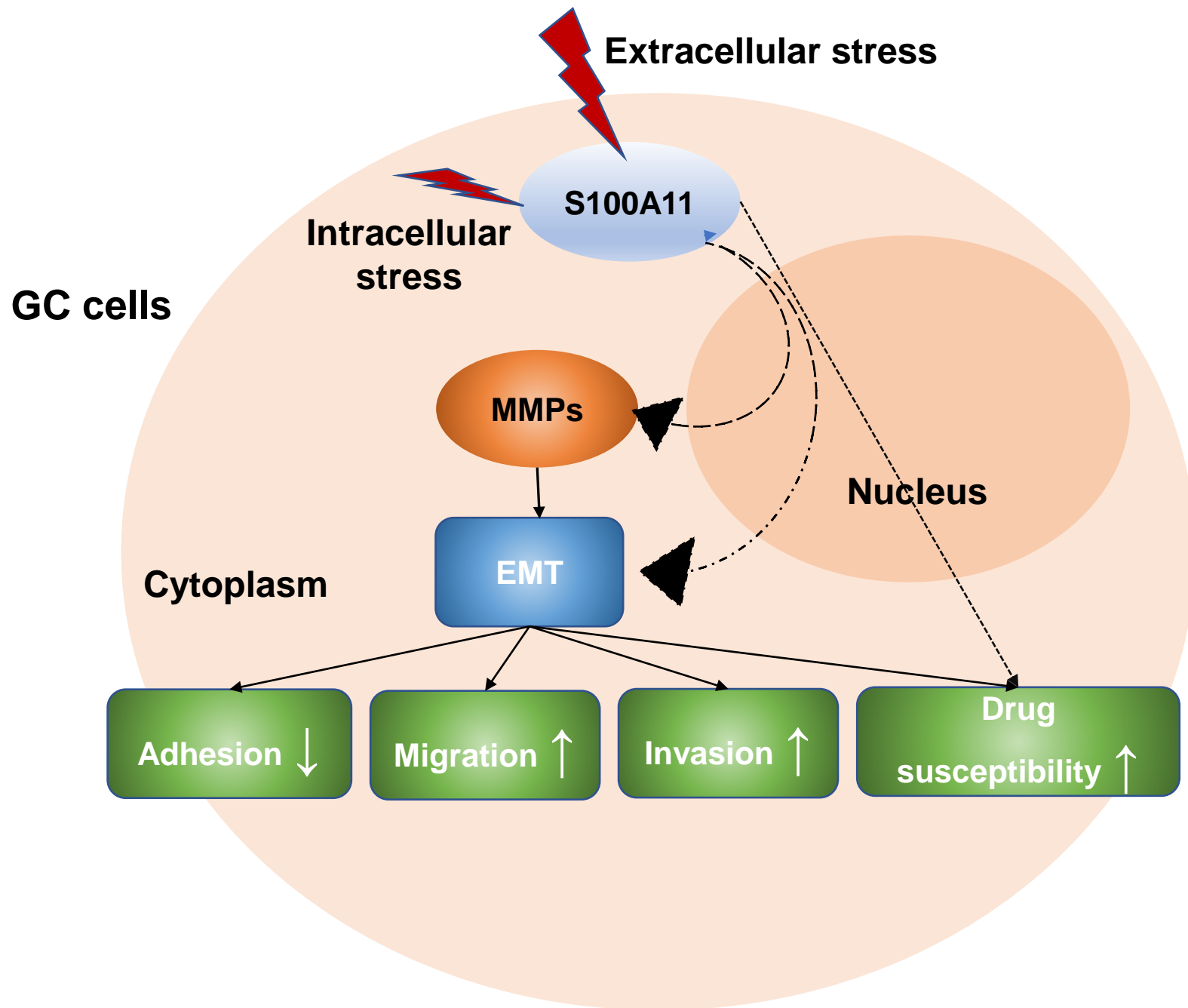

Supplement: Supplementary file 3 — Additional file 3: Figure S3. Schematic illustration of the functions of S100A11 in gastric cancer cells. [file 12935_2021_1949_MOESM3_ESM.pdf]
