# Supplementary material for: Dual effects of targeting S100A11 on suppressing cellular metastatic properties and sensitizing drug response in gastric cancer
Source: Cancer Cell Int. 2021 Apr 30;21:243. doi: 10.1186/s12935-021-01949-1 (PMC8086328; doi:10.1186/s12935-021-01949-1)
Supplement: Supplementary file 4 — Additional file 4: Table S1. Sequences of primer pairs for RT-PCR. [file 12935_2021_1949_MOESM4_ESM.docx]

**Supplementary Table 1. Sequences of primer pairs for RT-PCR.** For real-time qRT-PCR, a universal Z-sequence (ACTGAACCTGACCGTACA) was added to the 5'-end of the reverse primer, which is specific to a probe containing a 5’ hairpin structure labelled with a fluorophore (FAM).

| **Gene** | **Accession ID** | **RT-PCR type** | **Forward primer** | **Reverse primer** |
| --- | --- | --- | --- | --- |
| S100A11 | NM_005620 | Conventional | GTCCCTGATTGCTGTCTTCC | ACCAGGGTCCTTCTGGTTCT |
| S100A11 | NM_005620 | Real-time | GTATGCTGGAAAGGATGGTT | ACTGAACCTGACCGTACAAGGCAGCTAGTTCTGTATTC |
| E-cad | NM_004360 | Conventional | CGAGAGCTACACGTTCAC | GGGAAAAATAGGCTGTCCTT |
| N-cad | NM_001792 | Conventional | CAACGACGGGTTAGTCAC | ATTGGGGTCTGGAGTTTC |
| Snail | NM_005985 | Conventional | CGCTCTTTCCTCGTCAG | GTTGCAGTATTTGCAGTTGA |
| Slug | NM_003068 | Conventional | CTCTCCTCTTTCCGGATACT | AGCAGTTTTTGCACTGGTAT |
| Vimentin | NM_003380 | Conventional | GATGCTTCAGAGAGAGGAAG | CTCTTCGTGGAGTTTCTTCA |
| MMP2 | NM_004530 | Conventional | TTTGATGACGATGAGCTATG | TGCAGCTCTCATATTTGTTG |
| MMP3 | NM_002422 | Conventional | TCATTTTGGCCATCTCTTCC | GTGCCCATATTGTGCCTTCT |
| MMP9 | NM_004994 | Conventional | AACTACGACCGGGACAAG | ATTCACGTCGTCCTTATGC |
| GAPDH | NM_002046 | Conventional | AGGTCGGAGTCAACGGATTTG | GTGATGGCATGGACTGTGGT |
| GAPDH | NM_002046 | Real-time | CTGAGTACGTCGTGGAGTC | ACTGAACCTGACCGTACACAGAGATGATGACCCTTTTG |
